# Self Reports of Day-to-Day Function in a Small Cohort of People with Prodromal and Early HD

**DOI:** 10.1371/currents.RRN1254

**Published:** 2011-08-11

**Authors:** Janet Williams, Nancy Downing, Anthony L Vaccarino, Mark Guttman, Jane S. Paulsen

**Affiliations:** ^*^College of Nursing, The University of Iowa, Iowa City, IA; ^‡^Research Methods, Ontario Cancer Biomarker Network, Toronto, Ontario, Canada; ^§^Division of Neurology, Department of Medicine, University of Toronto, Toronto, Ontario Canada and ^¶^Department of Psychiatry, The University of Iowa Carver College of Medicine, Iowa City, IA, USA

## Abstract

Day-to-day functioning is a component of health-related quality of life and is an important end point for therapies to treat Huntington Disease (HD). Specific areas of day-to-day function changes have not been reported for prodromal or very early stages of HD. An exploratory self-report telephone interview was conducted with sixteen people with prodromal HD or early HD who met criteria designed to capture research participants most near to motor diagnosis. All completed semi-structured interviews on function in nine aspects of day-to-day life. Out of 16, 14 reported changes in at least one area. All day-to-day function areas were endorsed by at least one participant with driving being the most common area endorsed by 11/16. Changes in ability to perform some day-to-day tasks are experienced by people who are close to the time of clinical diagnosis for HD. Functional ability is likely to be an important component of outcome assessments of clinical trials and in ongoing clinical management.

## **Introduction**

Huntington disease (HD) is an autosomal dominant, progressive neurodegenerative disorder in which losses of neurologic function continue until the end of the person’s life. The diagnosis of HD traditionally is made with onset of motor symptoms. This typically occurs in the fourth decade of life [1], when individuals are employed and may be parenting minor age or young adult children. Cognitive, motor, sensory, physiologic, and neuroimaging markers of the earliest phase of this condition have been reported [2], but clear descriptions of the effects of these changes on day-to-day function have not yet been possible due to limited sensitivity of existing measures [2]. 

In plain language, function refers to a person’s abilities to do their daily tasks and routines in their daily life. However, the concept of ability to perform daily tasks is used in a variety of ways. The FDA description of patient-reported outcomes in clinical trials includes measures of any aspect of a patient’s health status that document the effect of a disease on a person’s quality of life, which in turn may include the person’s ability to perform daily activities (FDA, 2006). In the public health arena, health related quality of life refers to the effect of chronic illness on a person’s day-to-day life [3]. The notion of function status is a component of a health-related quality of life model [4], and it refers to the ability to perform tasks in multiple domains such as one’s physical, social role, and psychological function on a day-to-day basis [5]. 

The earliest phase described in HD is referred to as the prodrome, and research findings have documented features of the disease up to 15 years prior to the diagnosis [2]. Ongoing studies in the US and in Europe on prodromal HD (PREDICT-HD and TRACK-HD) [6] [7] use a variety of measures of functional assessments, including the UHDRS TFC scale [8], SF 36 [9], and Functional Assessment scale (FAS) [10]. However, these measures do not provide sufficient detail regarding specific functional deficits, nor do they assess factors that may contribute to these changes. The UHDRS TFC scale [8] is used in TRACK-HD and PREDICT-HD. In addition, TRACK-HD uses participant self reports on the SF 36 [7]. The SF 36 provides scales to measure physical and social aspects of functioning [9]. The TFC includes ratings by the person with prodromal HD or with the person’s companion, of a person’s self reported abilities to maintain their occupation, finances, domestic chores, activities of daily living, and whether the person resides at home with or without support or in a full time skilled nursing facility. The FAS [10] is a questionnaire of tasks related to occupation, finances, activities of daily living, domestic chores, level of care, and physical abilities. 

These global measures of functioning may not be sensitive to subtle changes in functional abilities that occur in prodromal HD. For example, when administered to 786 participants in PREDICT-HD, over 88% of participants scored at ceiling on the TFC or FAS. Among those participants, 5–7% reported some loss on questions about work and managing finances [2]. A separate analysis of 265 people who were diagnosed as having HD during their prospective participation in Huntington Study Group projects [11] identified that functional loss is strongly related to changes in motor scores; cognitive scores were also associated with decreased function regarding managing finances, driving safely, supervising children, and being able to volunteer; depression scores were related to loss of ability to engage in usual employment. Thus, measures to be used in upcoming clinical trials and in clinical assessments of people with prodromal HD would be needed that are sensitive to day-to-day real-life function. Although measures are under development to address specific components of function, including one’s work performance and quality of life [12], no measure captures the full range of tasks that comprise the day-to-day life of people with prodromal HD.

Research on the potential effect of interventions to delay or modify symptoms is limited by the absence of measures of day-to-day function activities across the prodromal and early diagnostic phases of HD. This includes those activities that may be diminishing at the time surrounding the time of diagnosis. Instruments are needed that document patient reported outcomes [13]. When developing these measures, qualitative methods such as interviews may be useful to identify relevant and appropriately worded items [14]. The purpose of this study was to systematically examine the range of day-to-day tasks and limits on the performance of day-to-day tasks reported by people with prodromal HD or recently diagnosed HD, in order to more completely characterize changes experienced in this time period for the development of patient reported outcome measures.

As reviewed above, the available quantitative data has been queried with regards to day-to-day functions in prodromal and very early HD. Findings have suggested that the currently available measures lack sensitivity to prodromal and very early diseases. This limitation is expected since most functional scales were initially developed for neurodegenerative diseases to track stages of dementia. Only more recently have we had the ability to identify and track persons at high risk for neurodegenerative diseases through efforts such as Mild Cognitive Impairment and PREDICT-HD. Findings suggest that new information is needed to better develop the earliest functional changes that occur prior to formal diagnosis of neurodegenerative disease. The current study was designed to solicit qualitative data to assist with the development of more sensitive measures. In an effort to maximize the data obtained through the qualitative interviews, we created an algorithm to recruit prodromal research volunteers who are considered most close to receiving a formal motor diagnosis and who were most likely to experience early functional decline. 

## **Methods**

This is an exploratory study using descriptive qualitative methods and a single telephone semi-structured interview. 

### Participants

Sixteen people who were enrolled at one of two PREDICT-HD research sites participated in the IRB-approved telephone interviews. Participants were selected from the PREDICT-HD study based on one of the following criteria:

(1) were considered to be “near” diagnosis based upon formula derived from CAG repeat length and current age [15]; (2) received a motor diagnosis according to the UHDRS motor score within the last 12 months; (3) obtained a UHDRS total motor score of >10 at their most recent PREDICT-HD visit; or (4) were in the 75^th^ percentile of longitudinal change in UHDRS total motor score. 

The project received approval from The University of Iowa Institutional Review Board and the Centre for Addiction and Mental Health Research Ethics Board in Toronto, Canada. The majority of participants (11/16) were female. Six were from Canada and 10 from the US. The mean CAG length was 41.38 (SD 1.45) and range 38–44. Mean age was 65.6 (SD 10.0) and range 36.5–78.8. Twelve had been diagnosed at the time of the interview.

### Interview Guide and Data Collection

A semi-structured interview guide (Figure 1) was developed by the authors for this project. Topics were selected based on a review of the literature, clinical experience of the research team, and prior interviews conducted during the development of an instrument focused specifically on ability to perform tasks at work. After completion of informed consent procedures, each person participated in a telephone interview conducted by a team member who is an experienced interviewer. Interviews ranged from 4–35 minutes, with the average being 13.5 minutes. The shorter interview times were for those participants who had no functional changes to report. All interviews were audiotaped and transcribed verbatim. Data saturation was reached after six interviews; after that we did not learn of other day-to-day function changes not previously mentioned by other participants.

**Figure 1. Day-to-Day Function Semi-Structured Interview Guide**

**Table:** 

We are interested in learning how things are going in your day-to-day life. What is this like for you? Some people have told us they have noticed changes in their ability to do certain day-to-day tasks. I’ll ask you about some specific topics. [Probes for each question] In what areas do you need help, What do you do about this, Has anyone commented on your (fill in the topic)? 1. Please tell me about your household chores. Do you ordinarily take care of your own home? What tasks do you ordinarily do? Have there been any changes in your ability to do these chores? 2. Please tell me about getting to places you need to go. Do you ordinarily drive or use public transportation? Do you usually get around by yourself? Has there been a change in this? Have you stopped doing things because of problems getting around? 3. Please tell me about shopping. Do you shop for things like groceries, items for your home, yourself, your car? 4. Please tell me about cooking and meals at home. Do you ordinarily prepare meals at home? 5. Please tell me about using the telephone. Do you ordinarily make calls, answer the phone? 6. Please tell me about things you usually enjoy. Do you start these hobbies on your own? Do you enjoy them if someone else gets you started or takes you where you can enjoy that activity, e.g., going to church, going fishing, bowling? 7. Please tell me about your ability to handle your finances. Do you ordinarily take care of these yourself? 8. Please tell me about work. Do you ordinarily work for pay; is this full time work? 9. Please tell me about being around, or interacting with, other people: In what areas do you see changes, e.g., avoid being around other people, become irritated or impatient with people 10. Last question: What else would you like to tell me?

## **Analysis**

Two authors followed qualitative descriptive analysis techniques [16]. The data were initially coded using NVivo 8 software [17]. Data were independently coded and reviewed until there was 100% agreement between the two authors.

## **Results**

Fourteen of the 16 participants described one or more changes in performance of day-to-day tasks. Each participant reporting changes endorsed from 1–7 areas of function (Table 1). One person who did not endorse any of the 9 topics in the interview guide reported the presence of involuntary movements. Specific functional tasks and examples of statements are provided below. Participants are identified by a number only for the purpose of reporting the data. 

**Table 1. Functional Changes (N=16)**

**Table:** 

Driving	11
Interactions	8
Household chores	8
Telephone	7
Shopping	6
Finances	5
Paid work	4
Cooking	4
Hobbies	1
Other-Motor	1

****

**Driving: ** The most commonly endorsed day-to-day change was driving (11/16). Participants referred to concerns about doing more than one thing at a time, being slower, and concerns about safety. For example, *I can only, kind of, do one thing at a time now, I need to be alert to the driving solely; my reaction time is slower (1), * and *one of the kids was nervous of me driving. Because of that I have said that I won’t drive any of the grandkids (2).*

**Interactions: ** When describing changes in interactions with others, participants described a variety of changes including lack of interest, anxiety, and irritability with others. *I’m, um, a little less patient, sometimes I have to watch myself, you know; I have to watch the way I speak (3),* and * it’s not that I don’t feel social, it’s just . . . I get lazy and I don’t want to go out (4).*

**Household chores: ** Respondents were aware they were not keeping up with their usual responsibilities, but described a lack of interest or initiative. *I used to change the beds and now it’s not as often as I used to (5), You know, I could look at something, and you know, realize it was dusty, but . . . for that to register in my head that I, you know, had to clean a whole house . . . I just never thought about it (6), * and *it’s not important any more (7).*

**Telephone: ** Respondents were reluctant to respond on the telephone when they weren’t prepared, and were selective when they did answer the telephone. * Only if I know about it in advance. Spur of the moment . . . forget it . . . it’s like because it was unexpected . . . my brain’s not working that way (7), *and* I have it print out on my TV so I it tells me who it is . . . I don’t want to get into the conversation . . . you just don’t want to . . . initiate it (8).*

**Shopping: ** Skills needed for shopping included being able to remember where to go for needed items, as well as what the items are. *I make my list, and write it down . . . to make sure I remember to stop (8), * and* I forget things so I usually need to have a list for everything I’m going to need (1).*

**Finances: ** Not being able to remember to take care of finances was mentioned by several; others also noted that they could not keep up with the processes of calculating and managing them. *Anything concerning the bills, or anything like that, I can’t remember (3), uh, I just kind of don’t do it, I mean, I don’t know what happened (6), I’m not very good at it, you know, adding in my head anymore (5).*

**Paid work: ** All respondents who left paid employment had done so voluntarily. Some stated they were able to retire due to having reached the requirements for full retirement, or for family reasons. Others specifically addressed an inability to do their jobs. *I had to leave my job because . . . I just couldn’t work, like I just couldn’t . . . mentally . . . I couldn’t do it (3). I was falling behind (6).*

**Cooking: ** Multiple demands in planning and executing preparing a meal were difficult for some participants. *I think I make more of a mess . . . But for the most part it’s ok; it takes me longer to clean up . . . I just forget to do things (4).*

**Hobbies and other topics: ** When asked about changes in hobbies or leisure activities, none of the respondents reported changes that were related to having prodromal HD. When asked if there were other things they had noticed, comments were offered about motor skills. *Oh God, that’s bad too; I walk into walls (7), I guess what you call the involuntary movements . . . I definitely feel that it doesn’t mean it makes any changes in your ability to do things . . . it’s just something you have noticed over time, that’s all (9).*

**

### Comments about Functional Changes

In responding to queries regarding potential changes in ability to carry out day-to-day tasks in specific areas, all respondents provided information on factors that, they believed, made it difficult to do these tasks. These factors ranged from emotional to cognitive to physical stamina topics. 

**Apathy/Fatigue:** *I get really tired . . . It’s not so much a physical thing; it’s a, like in your mind (10). I don’t have the excitement part of doing some things; it’s just not there (11).*

**Cognition:** *Part of my trade was . . . always learning stuff; I couldn’t learn . . . I couldn’t remember anything anymore (4); Memory—it’s just gone. Like my short-term memory (3).*

**Irritability:** *I always seem to have that real quick to anger (6). I’m a little short, a little cranky (12).*

**

## **Discussion**

This study examined self-reports of losses of abilities to perform day-to-day tasks in a small cohort of people in the prodromal HD or very early phase of HD and for whom scores on the UHRDS motor scale indicated a significant increase of motor decline. Findings indicate that changes in day-to-day task function vary in prodromal HD and those recently diagnosed with HD, but that when changes are experienced, they are more likely to be in the domains of tasks that require multiple cognitive, motor, and behavioral abilities. Driving is a good illustration of this because it is an activity that requires multiple domain skills and was the most frequently mentioned area of functional difficulty in this sample. Although few reports focus on components of day-to-day function in prodromal HD or early HD, the range of topics is consistent with those reported with less sensitive measures, specifically that of employment and managing finances [2], which are endorsed by this cohort. In a study of changes in people with prodromal HD over a 12 month period, changes in function as measured by the TFC are reported to show greater change than controls, but this did not reach statistical significance [18]. 

      A potential limitation of this study includes the possibility of early lack of insight that is a component of HD [19] that has also been found in persons with prodromal HD. As people with prodromal HD approached diagnosis, increasing discrepancies between self-ratings and companion ratings of apathy, disinhibition, and executive function have been documented [20]. On the other hand, self-report of cognitive impairment has been demonstrated to be a reliable indicator of mild cognitive impairment in studies of people in early stages of dementia [21] [22]. 

      These findings suggest that day-to-day function may be impaired in some domains during the prodromal HD and recently diagnosed period, and that measures to document function must encompass the range of domains likely to be affected in prodromal HD. Data from these interviews were used in the development of items to assess day-to-day functioning by FuRST-pHD; and initiative to develop a rating scale for assessing symptoms and functional ability in prodromal and early HD (http://knol.google.com/k/anthony-l-vaccarino/assessment-of-day-to-day-functioning-in/19jerwgzmryar/28#). Further investigations may provide insight into factors associated with the domains in which people with prodromal HD perceive changes, as well as provide data to monitor progression of day-to-day function over time.  

## **Funding information** 

This research is supported by the National Institutes for Health, National Institute of Neurological Disorders and Stroke (NS40068) and CHDI Foundation, Inc. 

## **Acknowledgments** 

We thank the PREDICT-HD sites, the study participants, and the National Research Roster for Huntington Disease Patients and Families. The full list of those involved with the PREDICT-HD study is shown below.

## **Competing interests** 

The authors declare no competing interests.

**PREDICT-HD Investigators, Coordinators, Motor Raters, Cognitive Raters**

*Active: September 2009–August 2010*

Thomas Wassink, MD, Stephen Cross, BA, Nicholas Doucette, BA, Mycah Kimble, BA, Patricia Ryan, MSW, LISW, MA, Jessica Wood, MD, PhD, Eric A. Epping, MD, PhD, and Leigh J. Beglinger, PhD (University of Iowa, Iowa City, Iowa, USA); Edmond Chiu, MD, Olga Yastrubetskaya, PhD, Joy Preston, Anita Goh, D.Psych, Chathushka Fonseka, and Liz Ronsisvalle (St. Vincent’s Hospital, The University of Melbourne, Kew, Victoria, Australia); Phyllis Chua, MD, and Angela Komiti, BS, MA (The University of Melbourne, Royal Melbourne Hospital, Melbourne, Australia); Lynn Raymond, MD, PhD, Rachelle Dar Santos, BSc, and Joji Decolongon, MSC, CCRP (University of British Columbia, Vancouver, British Columbia, Canada); Adam Rosenblatt, MD, Christopher A. Ross, MD, PhD, Barnett Shpritz, BS, MA, OD, and Claire Welsh (Johns Hopkins University, Baltimore, Maryland, USA); William M. Mallonee, MD, Greg Suter, BA, and Judy Addison (Hereditary Neurological Disease Centre, Wichita, Kansas, USA); Ali Samii, MD, and Alma Macaraeg, BS (University of Washington and VA Puget Sound Health Care System, Seattle, Washington, USA); Randi Jones, PhD, Cathy Wood-Siverio, MS, Stewart A. Factor, DO, and Claudia Testa, MD, PhD (Emory University School of Medicine, Atlanta, Georgia, USA); Roger A. Barker, BA, MBBS, MRCP, Sarah Mason, BSC, Anna Goodman, PhD, Rachel Swain, BA, and Anna DiPietro (Cambridge Centre for Brain Repair, Cambridge, UK); Elizabeth McCusker, MD, Jane Griffith, RN, Clement Loy, MD, David Gunn, BS, and Linda Stewart, RN (Westmead Hospital, Sydney, Australia); Bernhard G. Landwehrmeyer, MD, Michael Orth MD, PhD, Sigurd Süβmuth, MD, RN, Katrin Barth, RN, and Sonja Trautmann, RN (University of Ulm, Ulm, Germany); Kimberly Quaid, PhD, Melissa Wesson, MS, and Joanne Wojcieszek, MD (Indiana University School of Medicine, Indianapolis, IN); Mark Guttman, MD, Alanna Sheinberg, BA, and Irita Karmalkar, BSc (Centre for Addiction and Mental Health, University of Toronto, Markham, Ontario, Canada); Susan Perlman, MD and Arik Johnson, PsyD (University of California, Los Angeles Medical Center, Los Angeles, California, USA); Michael D. Geschwind, MD, PhD, Jon Gooblar, BA, and Gail Kang, MD (University of California San Francisco, California, USA); Tom Warner, MD, PhD, Maggie Burrows, RN, BA, Marianne Novak, MD,** **Thomasin Andrews, MD, BSC, MRCP, Elisabeth Rosser, MBBS, FRCP, and Sarah Tabrizi, MD, PhD (National Hospital for Neurology and Neurosurgery, London, UK); Anne Rosser, MD, PhD, MRCP, Kathy Price, RN, and Sarah Hunt, BSc (Cardiff University, Cardiff, Wales, UK); Frederick Marshall, MD, Amy Chesire, LCSW-R, MSG, Mary Wodarski, BA, and Charlyne Hickey, RN, MS (University of Rochester, Rochester, New York, USA); Oksana Suchowersky, MD, FRCPC, Sarah Furtado, MD, PhD, FRCPC, and Mary Lou Klimek, RN, BN, MA (University of Calgary, Calgary, Alberta, Canada); Peter Panegyres, MB, BS, PhD, Elizabeth Vuletich, BSC, Steve Andrew, and Rachel Zombor, MPSYC (Neurosciences Unit, Graylands, Selby-Lemnos & Special Care Health Services, Perth, Australia); Joel Perlmutter, MD, Stacey Barton, MSW, LCSW, and Amy Schmidt (Washington University, St. Louis, Missouri, USA); Zosia Miedzybrodzka, MD, PhD, Sheila A. Simpson, MD, Daniela Rae, RN, and Mariella D’Alessandro, PhD (Clinical Genetics Centre, Aberdeen, Scotland, UK); David Craufurd, MD, Ruth Fullam, BSC, and Elizabeth Howard, MD (University of Manchester, Manchester, UK); Pietro Mazzoni, MD, PhD, Karen Marder, MD, MPH, and Paula Wasserman, MA (Columbia University Medical Center, New York, New York, USA); Rajeev Kumar, MD and Diane Erickson, RN (Colorado Neurological Institute, Englewood, Colorado, USA); Vicki Wheelock, MD, Terry Tempkin, RNC, MSN, Nicole Mans, BA, MS, and Kathleen Baynes, PhD (University of California Davis, Sacramento, California, USA); Joseph Jankovic, MD, Christine Hunter, RN, CCRC, and William Ondo, MD (Baylor College of Medicine, Houston, Texas, USA); Justo Garcia de Yebenes, MD, Monica Bascunana Garde, Marta Fatas, BA,  and Asuncion Martinez-Descales (Hospital Ramón y Cajal, Madrid, Spain); Wayne Martin, MD, Pamela King, BScN, RN, and Satwinder Sran, BSC (University of Alberta, Edmonton, Alberta, Canada); Anwar Ahmed, PhD, Stephen Rao, PhD, Christine Reece, BS, Janice Zimbelman, PhD, PT, Alexandra Bea, BA, Emily Newman, BA, and Alex Bura, BA (Cleveland Clinic Foundation, Cleveland, Ohio, USA).

**Steering Committee**

Jane Paulsen, PhD, Principal Investigator, Eric A. Epping, MD, PhD, Hans Johnson, PhD, Megan Smith, PhD, Janet Williams, PhD, RN, FAAN, Leigh Beglinger, PhD, James Mills, MS (University of Iowa Hospitals and Clinics, Iowa City, IA); Elizabeth Aylward, PhD (Seattle Children's Research Institute, WA); Kevin Biglan, MD (University of Rochester, Rochester, NY); Blair Leavitt, MD (University of British Columbia, Vancouver, BC, Canada); Marcy MacDonald, PhD (Massachusetts General Hospital); Martha Nance, MD (Hennepin County Medical Center, Minneapolis, MN); and Cheryl Erwin, JD, PhD (University of Texas Medical School at Houston).

**Scientific Sections**

**Bio Markers: **Blair Leavitt, MDCM, FRCPC (Chair) and Michael Hayden, PhD (University of British Columbia); Stefano DiDonato, MD (Neurological Institute “C. Besta,” Italy); Ken Evans, PhD (Ontario Cancer Biomarker Network); Wayne Matson, PhD (VA Medical Center, Bedford, MA); Asa Peterson, MD, PhD (Lund University, Sweden), Sarah Tabrizi, MD, PhD (National Hospital for Neurology and Neurology and Neurosurgery, London); Beth Borowsky, PhD (CHDI); Andrew Juhl, BS, James Mills, MS, Kai Wang, PhD (University of Iowa); and David Weir, BSc (University of British Columbia).

**Brain:** Jean Paul Vonsattell, PhD (Chair), and Carol Moskowitz, ANP, MS (Columbia University Medical Center); Anne Leserman, MSW, LISW, Lynn Schaul, BA, and Stacie Vik, BA (University of Iowa).

**Cognitive:** Deborah Harrington, PhD (Chair), Gabriel Castillo, BS, Jessica Morison, BS, and Jason Reed, BS (University of California, San Diego), Michael Diaz, PhD, Ian Dobbins, PhD, Tamara Hershey, PhD, Erin Foster, OTD, and Deborah Moore, BA (Washington University Cognitive Science Battery Development); Holly Westervelt, PhD (Chair, Quality Control and Training, Alpert Medical School of Brown University), Jennifer Davis, PhD, and Geoff Tremont, PhD, MS (Scientific Consultants, Alpert Medical School of Brown University); Megan Smith, PhD (Chair, Administration), David J. Moser, PhD, Leigh J. Beglinger, PhD, Kelly Rowe, and Danielle Theriault, BS (University of Iowa); Carissa Gehl, PhD (VA Medical Center, Iowa City, IA); Kirsty Matheson (University of Aberdeen); Karen Siedlecki, PhD (Fordham University); Marleen Van Walsem (EHDN); Susan Bonner, BA, Greg Elias, BA, and Melanie Faust, BS (Rhode Island Hospital); Beth Borowski, PhD (CHDI); Noelle Carlozzi (University of Michigan); Kevin Duff, PhD (University of Utah); Nellie Georgiou-Karistianis (St. Vincent’s Hospital, The University of Melbourne, Australia); Julie Stout, PhD (Monash University, Melbourne, Australia); Herwig Lange (Air-Rahazentrum); and Kate Papp (University of Connecticut).

**Functional**: Janet Williams, PhD (Chair), Leigh J. Beglinger, PhD, Anne Leserman, MSW, LISW, Eunyoe Ro, MA, Lee Anna Clark, Nancy Downing, RN, PhD, Joan Laing, PhD, Kristine Rees, BA, and Stacie Vik, BA (University of Iowa); Rebecca Ready, PhD (University of Massachusetts); Anthony Vaccarino, PhD (Ontario Cancer Biomarker Network); Sarah Farias, PhD (University of California, Davis); Noelle Carlozzi, PhD (University of Michigan); and Carissa Gehl, PhD (VA Medical Center, Iowa City, IA).

**Genetics:** Marcy MacDonald, PhD (Co-Chair), Jim Gusella, PhD, and Rick Myers, PhD (Massachusetts General Hospital); Michael Hayden, PhD (University of British Columbia); Tom Wassink, MD (Co-Chair) Eric A. Epping, MD, PhD, Andrew Juhl, BA, James Mills, MS, and Kai Wang, PhD (University of Iowa); Zosia Miedzybrodzka, MD, PhD (University of Aberdeen); and Christopher Ross, MD, PhD (Johns Hopkins University).

**Imaging:** *Administrative:* Ron Pierson, PhD (Chair), Kathy Jones, BS, Jacquie Marietta, BS, William McDowell, AA, Greg Harris, BS, Eun Young Kim, MS, Hans Johnson, PhD, and Thomas Wassink, MD (University of Iowa); John Ashburner, PhD (Functional Imaging Lab, London); Steve Potkin, MD (University of California, Irvine); and Arthur Toga, PhD (University of California, Los Angeles). *Striatal:* Elizabeth Aylward, PhD (Chair, Seattle Children's Research Institute). *Surface Analysis:* Eric Axelson, BSE (University of Iowa). *Shape Analysis:* Christopher A. Ross (Chair), MD, PhD, Michael Miller, PhD, and Sarah Reading, MD (Johns Hopkins University); Mirza Faisal Beg, PhD (Simon Fraser University). *DTI: *Vincent A. Magnotta, PhD (Chair, University of Iowa); Karl Helmer, PhD (Massachusetts General Hospital); Kelvin Lim, MD (University of Ulm, Germany); Mark Lowe, PhD (Cleveland Clinic); Sasumu Mori, PhD (Johns Hopkins University); Allen Song, PhD (Duke University); and Jessica Turner, PhD (University of California, Irvine). *fMRI:* Steve Rao, PhD (Chair), Erik Beall, PhD, Katherine Koenig, PhD, Michael Phillips, MD, Christine Reece, BS, and Jan Zimbelman, PhD, PT (Cleveland Clinic); and April Bryant (University of Iowa).

**Motor:** Kevin Biglan, MD (University of Rochester), Karen Marder, MD (Columbia University), and Jody Corey-Bloom, MD, PhD (University of California, San Diego) all Co-Chairs; Michael Geschwind, MD, PhD (University of California, San Francisco); Ralf Reilmann, MD and Zerka Unds (Muenster, Germany); and Andrew Juhl, BS (University of Iowa).

**Psychiatric:** Eric A. Epping, MD, PhD (Chair), Nancy Downing, RN, PhD, Jess Fiedorowicz, MD, Robert Robinson, MD, Megan Smith, PhD, Leigh Beglinger, PhD, James Mills, MS, Kristine Rees, BA, Adam Ruggle, Stacie Vik, BA, Janet Williams, PhD, Dawei Liu, PhD, David Moser, PhD, and Kelly Rowe (University of Iowa); Karen Anderson, MD (University of Maryland); David Craufurd, MD (University of Manchester); Mark Groves, MD (Columbia University); Anthony Vaccarino, PhD and Ken Evans, PhD (Ontario Cancer Biomarker Network); Hugh Rickards, MD (Queen Elizabeth Psychiatric Hospital); Eric van Duijn, MD (Leiden University Medical Center, Netherlands); Irina Antonijevic, MD, PhD, and Joseph Giuliano (CHDI); Phyllis Chua (The University of Melbourne, Royal Melbourne Hospital); and Kimberly Quaid, PhD (Indiana University School of Medicine).

**Core Sections**

**Statistics:** James Mills, MEd, MS, Dawei Liu, PhD, Jeffrey Long, PhD, Wenjing Lu, Kai Wang, PhD, and Ying Zhang, PhD (University of Iowa).

**Recruitment/Retention:** Martha Nance, MD (Chair, University of Minnesota); Anne Leserman, MSW, LISW, Nicholas Doucette, BA, Mycah Kimble, BA, Patricia Ryan, MSW, LISW, MA, Kelli Thumma, BA, Elijah Waterman, BA, and Jeremy Hinkel, BA (University of Iowa).

**Ethics:** Cheryl Erwin, JD, PhD, (Chair, McGovern Center for Health, Humanities and the Human Spirit); Eric A. Epping, MD, PhD Janet Williams, PhD, Nicholas Doucette, BA, Anne Leserman, MSW, LISW, James Mills, MS, Lynn Schaul, BA, and Stacie Vik, BA (University of Iowa); Martha Nance, MD (University of Minnesota); and Lisa Hughes, MEd (University of Texas Medical School at Houston).

**IT/Management:** Hans Johnson, PhD (Chair), R.J. Connell, BS, Karen Pease, BS, Ben Rogers, BA, BSCS, Jim Smith, AS, Shuhua Wu, MCS, Roland Zschiegner, Erin Carney, Bill McKirgan, Mark Scully, and Ryan Wyse (University of Iowa); Jeremy Bockholt (AMBIGroup).

**Program Management**

**Administrative:** **Chris Werling-Witkoske (Chair), Karla Anderson, BS, Kristine Bjork, BA, Ann Dudler, Jamy Schumacher, Sean Thompson, BA, Leann Davis, Machelle Henneberry, Greg Ennis, MA, and Stacie Vik, BA (University of Iowa).

**Financial:** **Steve Blanchard, MSHA, Kelsey Montross, BA, and Phil Danzer (University of Iowa).
